# Relationship between Quadriceps Tendon Young’s Modulus and Maximum Knee Flexion Angle in the Swing Phase of Gait in Patients with Severe Knee Osteoarthritis

**DOI:** 10.3390/medicina56090437

**Published:** 2020-08-28

**Authors:** Bungo Ebihara, Takashi Fukaya, Hirotaka Mutsuzaki

**Affiliations:** 1 Graduate School of Health Sciences, Ibaraki Prefectural University of Health Sciences, 4669-2 Ami, Ami-machi, Inashiki-gun, Ibaraki 300-0394, Japan; 2 Department of Rehabilitation, Tsuchiura Kyodo General Hospital, 4-1-1 Otsuno, Tsuchiura, Ibaraki 300-0028, Japan; 3 Department of Physical Therapy, Faculty of Health Sciences, Tsukuba International University, 6-8-33 Manabe, Tsuchiura, Ibaraki 300-0051, Japan; t-fukaya@tius.ac.jp; 4 Department of Orthopaedic Surgery, Ibaraki Prefectural University of Health Sciences, 4669-2 Ami, Ami-machi, Inashiki-gun, Ibaraki 300-0394, Japan; mutsuzaki@ipu.ac.jp

**Keywords:** quadriceps tendon, Young’s modulus, elastography, maximum knee flexion angle, gait, knee osteoarthritis

## Abstract

*Background and objectives*: Decreased knee flexion in the swing phase of gait can be one of the causes of falls in severe knee osteoarthritis (OA). The quadriceps tendon is one of the causes of knee flexion limitation; however, it is unclear whether the stiffness of the quadriceps tendon affects the maximum knee flexion angle in the swing phase. The purpose of this study was to clarify the relationship between quadriceps tendon stiffness and maximum knee flexion angle in the swing phase of gait in patients with severe knee OA. *Materials and Methods*: This study was conducted from August 2018 to January 2020. Thirty patients with severe knee OA (median age 75.0 (interquartile range 67.5–76.0) years, Kellgren–Lawrence grade: 3 or 4) were evaluated. Quadriceps tendon stiffness was measured using Young’s modulus by ShearWave Elastography. The measurements were taken with the patient in the supine position with the knee bent at 60° in a relaxed state. A three-dimensional motion analysis system measured the maximum knee flexion angle in the swing phase. The measurements were taken at a self-selected gait speed. The motion analysis system also measured gait speed, step length, and cadence. Multiple regression analysis by the stepwise method was performed with maximum knee flexion angle in the swing phase as the dependent variable. *Results*: Multiple regression analysis identified quadriceps tendon Young’s modulus (standardized partial regression coefficients [*β*] = −0.410; *p* = 0.013) and gait speed (*β* = 0.433; *p* = 0.009) as independent variables for maximum knee flexion angle in the swing phase (adjusted coefficient of determination = 0.509; *p* < 0.001). *Conclusions*: Quadriceps tendon Young’s modulus is a predictor of the maximum knee flexion angle. Clinically, decreasing Young’s modulus may help to increase the maximum knee flexion angle in the swing phase in those with severe knee OA.

## 1. Introduction

Knee osteoarthritis (OA) has a complex pathogenesis that involves mechanical, inflammatory, and metabolic factors, which leads to joint destruction, and causes disability [1]. Gait function decreases gradually with the increase in the severity of knee OA [2]. Moreover, patients with knee OA are more likely to fall. In fact, patients with severe knee OA are at a higher risk of falling compared to healthy older adults, and tripping is the main cause of falling [3]. A gait variable involved in tripping is foot clearance [4]; it was previously reported that toe clearance in fallers is less than that of non-fallers [5]. Toe clearance is affected by knee flexion in the swing phase [6]. Knee flexion is also essential for lifting the foot [7]. However, the maximum knee flexion angle in the swing phase is decreased in patients with severe knee OA [8]. Thus, a decreased knee flexion angle in the swing phase can be one of the causes of falls.

Slow gait speed reduces the maximum knee flexion angle in the swing phase [9], and it was reported that gait speed is slow in patients with severe knee OA [8]. In addition, in these patients, the quadriceps tendon limits their knee flexion range of motion (ROM) [10]. However, it is unclear whether the stiffness of the quadriceps tendon affects the maximum knee flexion angle in the swing phase.

ShearWave Elastography (SWE) is an imaging technique that aims to measure tissue stiffness. Tissue stiffness is generally measured using Young’s modulus, and SWE generates shear waves in the body and calculates Young’s modulus from their propagation speed [11]. An increased Young’s modulus indicates that the tissue is stiff. In the past decade, SWE has been used to evaluate musculoskeletal tissues [12]. It has been reported that tendon stiffness assessed using SWE is related to ROM. Ankle dorsiflexion ROM is associated with Achilles tendon stiffness [13]; therefore, using the quadriceps tendon Young’s modulus can clarify the relationship between the quadriceps tendon stiffness and the maximum knee flexion angle in the swing phase.

The purpose of this study was to clarify the relationship between quadriceps tendon stiffness and the maximum knee flexion angle in the swing phase of gait in patients with severe knee OA. We hypothesized that the maximum knee flexion angle would decrease as the quadriceps tendon Young’s modulus increased because a stiff quadriceps tendon limits knee flexion ROM. Clinically, if the quadriceps tendon Young’s modulus is a predictor of the maximum knee flexion angle, decreasing Young’s modulus may help to increase the maximum knee flexion angle in the swing phase in severe knee OA.

## 2. Materials and Methods

### 2.1. Participants

This study was conducted from August 2018 to January 2020. The screened patients were either outpatients or were admitted for knee OA surgery. The inclusion criteria were knee OA severity of Kellgren–Lawrence (KL) grade 3 or 4, and the ability to walk independently. An orthopedic surgeon classified the severity of knee OA according to the KL grade by reviewing radiographs [14]. The exclusion criteria were symptoms of central nervous system disease and dementia. The symptomatic knees of the patients with knee OA was used for all measurements. When both knees were symptomatic, the knee with the greatest restrictions in the swing phase was used for all measurements.

Thirty-one patients with severe knee OA were evaluated. Each participant provided written informed consent. We collected data on the participants’ age, sex, height, weight, body mass index (BMI), and femorotibial angle (FTA). Bodyweight was measured using a scale, and other data were obtained from patients’ medical records.

### 2.2. Ethics Statement

This study was approved by the ethics committees of Tsuchiura Kyodo General Hospital and Ibaraki Prefectural University of Health Sciences, (approval numbers 690 and e159, approval date May 11, 2018 and July 5, 2018, respectively) and was conducted in accordance with the Declaration of Helsinki. Each participant provided written informed consent.

### 2.3. Measurement of Knee ROM

Participants were assessed in a supine position on a bed. Knee ranges of extension and flexion were measured through active movements using goniometry, with a minimum value of 1°. Participants extended and flexed the knee as hard as possible. The knee angles were measured from the intersection of the line connecting the greater trochanter of the femur and lateral epicondyle of the femur, and the line connecting the fibular head and lateral malleolus of the fibula.

### 2.4. Measurement of Quadriceps Tendon Young’s Modulus

Quadriceps tendon Young’s modulus was measured by SWE using the Aixplorer ultrasound unit in conjunction with a 2–10 MHz linear transducer (Supersonic Imaging, Aix-en-Provence, France). We selected a preset musculoskeletal and knee setting. We set the SWE Opt, which is a function to adjust elastography resolution and penetration, to penetration mode. SWE provides the value of Young’s modulus in kPa, with a minimum value of 0.1 kPa and a maximum value of 800 kPa. SWE was performed in a quiet private room, and the room temperature was controlled at 25 °C to reduce the effects of temperature on the tendon [15]. All ultrasound measurements were performed by the same physical therapist who had 2 years of experience in performing musculoskeletal SWE.

This measurement was taken without a break after walking to the examination room. The measurements were taken with the patient in the supine position with the knee bent at 60° because it is reported that the maximum knee flexion angle is approximately 60° in the swing phase in normal gait [7]. We placed towels and cushions under the participant’s knees to keep them relaxed. During the measurements, verbal instruction was given to the participants to refrain from talking, remain relaxed, and to avoid any muscle contraction.

**Figure 1:**
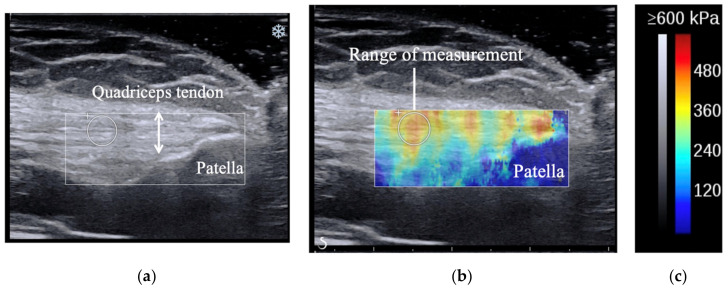
SWE images showing the positions of the patella, quadriceps tendon, and the range of measurements. The upper and lower ends of the arrows in (**a**) indicate the quadriceps tendon. The circle in (**b**) indicates the measurement range of Young’s modulus, and the stiffness is shown in blue to red according to Young’s modulus values. (**c**) Presents the gray and color scale.

The measurement procedure was mainly based on that described in a previous study, in which the intra-rater reliability of the measurement was reported to be good [16]. First, we placed the transducer between the muscle–tendon transition region of the rectus femoris muscle and the center of the base of the patella. The muscle–tendon transition region was confirmed with an ultrasound image, and the center of the base of the patella was confirmed by palpation. Large amounts of gel were used between the skin and transducer to prevent transducer pressure to the participant’s tissue. Based on a study that evaluated the validity of SWE [17], the transducer was placed parallel to the quadriceps tendon fibers. The transducer was kept motionless for 5 to 10 s during the acquisition of the SWE sonogram video. The SWE sonogram video was converted to a still image when the quadriceps tendon sonogram image was stable. This still image was used to measure Young’s modulus. Based on a study that evaluated the repeatability of SWE [18], we measured the quadriceps tendon Young’s modulus 2 cm proximal to the bony insertion onto the patella. The range of measurements can include any size. We set the size as large as possible within the quadriceps tendon according to the thickness of the participants’ tendon. It is preferable to report mean values when using different sizes of range of measurement because the values at the range of interest were not uniform [19]. Therefore, we recorded the mean value. Typical examples of the SWE image are shown in Figure 1.

### 2.5. Measurement of Gait Parameters

To assess walking, kinematics data were obtained at 100 Hz using a 10-camera motion analysis system MA-3000 (Anima, Tokyo, Japan). The ground reaction force data were recorded at 100 Hz using four floor-mounted force plates MG-1060 (Anima, Tokyo, Japan), and the data were synchronized with the motion capture data. To put reflective markers directly above landmarks, participants were barefoot. Reflective markers 20 mm in diameter were placed directly over the following anatomical landmarks: the acromion, anterior superior iliac spine, greater trochanter, lateral femoral epicondyle, lateral malleolus, and the bone head of the fifth metatarsal bone. The marker position data and ground reaction forces were low-pass filtered at 10 Hz and 20 Hz, respectively.

The participants walked barefoot along a 6 m level walkway at a self-selected speed. We permitted participants to use one or two canes for fall prevention if necessary. The gait cycle was determined by the force plate data and movement speed of the reflective markers. The average of four gait trials was collected for each participant and used for analysis. We recorded gait speed, cadence, step length, and maximum knee flexion angle in the swing phase. Knee flexion angle was determined by the marker positions of the greater trochanter, lateral femoral epicondyle, and lateral malleolus. The above measurements of the gait cycle and gait parameters were performed by the software built into the three-dimensional motion analysis system.

### 2.6. Measurement of Pain during Gait

To evaluate pain during gait, we used the visual analog scale (VAS) [20]. A 100 mm self-filling linear scale was used. The markings were converted to a score between 0 and 100 by reading off each mark (no pain, 0 points; maximum pain, 100 points). This measurement was performed immediately after the measurement of gait parameters.

### 2.7. Statistical Analysis

A power analysis of the multiple regression using maximum knee flexion angle as the dependent variable was performed with an effect size of *f*^2^ = 0.35, an error probability of *α* = 0.05, a power of 0.8, and two predictors. The power analysis was performed using G*Power 3.1 [21]. This yielded an estimated sample size of 31 participants, which was thus included in this study.

The distribution of the data in this study was assessed by the Shapiro–Wilk’s test. Normally distributed data were calculated as mean and standard deviation values; otherwise median and interquartile range values were calculated.

Multiple regression analysis that used the stepwise method was performed with the maximum knee flexion angle in the swing phase as the dependent variable. The independent variables included measurement values that were significantly correlated with the maximum knee flexion angle in the swing phase, and included quadriceps tendon Young’s modulus, gait speed, cadence, and step length.

Correlations between measurement values were confirmed before multiple regression analysis. Because quadriceps tendon Young’s modulus and maximum knee flexion angle in the swing phase were not normally distributed, Spearman’s rank correlation coefficient was determined between the quadriceps tendon Young’s modulus and the other measurement values, and between the maximum knee flexion angle in the swing phase and the other measurement values.

*p*-values < 0.05 were considered statistically significant. SPSS statistics version 24.0 (IBM Corp., New York, NY, USA) was used to perform all the statistical analyses, except the power analysis.

## 3. Results

### 3.1. Participants’ Physical Characteristics and Measured Values

The quadriceps tendon Young’s modulus for one of the participants exceeded the upper limit of the SWE and was excluded from the study. The participant had osteophytes that extended from the patella to just below the measurement site. Therefore, the data of only 30 participants were described. The physical characteristics and measured values of the participants are summarized in Table 1 and Table 2, respectively.

### 3.2. Multiple Regression Analysis

The results of the multiple regression analysis are shown in Table 3. Quadriceps tendon Young’s modulus (*p* = 0.013) and gait speed (*p* = 0.009) were chosen as independent variables. The standardized partial regression coefficients (*β*) of the quadriceps tendon Young’s modulus and gait speed were −0.410 and 0.433, respectively. Cadence (*p* = 0.644) and step length (*p* = 0.747) were not chosen as independent variables. The multiple correlation coefficient was 0.737, the coefficient of determination (*R^2^*) was 0.543, and the adjusted coefficient of determination (adjusted *R^2^*) was 0.509. Multicollinearity was not observed between the independent variables (variance inflation factor (*VIF*) = 1.391). The Durbin–Watson ratio was 2.950 and the residuals were normally distributed (*p* = 0.589). The obtained prediction formula is shown below:

Maximum knee flexion angle in the swing phase (°) = 45.109 − 0.035 × quadriceps tendon Young’s modulus (kPa) + 22.367 × gait speed (m/s)(1)

### 3.3. Correlation Coefficients

The correlation results between the quadriceps tendon Young’s modulus and maximum knee flexion angle in the swing phase and the other measurement values are shown in Table 4. There were negative correlations between the quadriceps tendon Young’s modulus and the active flexion angle (*r_s_* = −0.557, *p* = 0.001), gait speed (*r_s_* = −0.426, *p* = 0.019), step length (*r_s_* = −0.508, *p* = 0.004), and the maximum knee flexion angle in the swing phase (*r_s_* = −0.559, *p* = 0.001). There were positive correlations between maximum knee flexion angle in the swing phase and gait speed (*r_s_* = 0.532, *p* = 0.002), cadence (*r_s_* = 0.503, *p* = 0.005), and step length (*r_s_* = 0.457, *p* = 0.011). There were no correlations between the other parameters (*p* > 0.05).

## 4. Discussion

This study showed that the quadriceps tendon Young’s modulus is related to the maximum knee flexion angle in the swing phase of gait in patients with severe knee OA. A prediction formula showed that the maximum knee flexion angle may decrease as the quadriceps tendon Young’s modulus increases. These findings support our hypotheses.

The quadriceps tendon Young’s modulus was a predictor of the maximum knee flexion angle in the swing phase in severe knee OA. Using SWE, it has been reported that there is a relationship between soft tissue stiffness and the ROM of joints [22,23]. The quadriceps tendon Young’s modulus of knee flexion at 60° of healthy adults was 227.2 ± 67.5 kPa [16]. If the quadriceps tendon Young’s modulus of knee flexion at 60° increases above the normal value, it may cause resistance and limit knee flexion movement, making it more difficult to bend the knee to the reported maximum knee flexion angle of 60° in the swing phase [7].

We also found that a decreased quadriceps tendon Young’s modulus may increase the maximum knee flexion angle in the swing phase. Elastography studies have reported that the degree of tendon stiffness changed with joint movement [16,24] and muscle contraction [25]. The quadriceps tendon Young’s modulus increased with knee flexion and/or quadriceps muscle contraction. As described previously, an increased Young’s modulus state indicated that the quadriceps tendon was stretched proximally or distally. Therefore, improving the flexibility of the quadriceps tendon and surrounding tissue or decreasing the quadriceps muscle tone may decrease the quadriceps tendon Young’s modulus.

There are five limitations to this study. First, there was a participant whose quadriceps tendon Young’s modulus could not be measured because it exceeded the upper limit of measurement. In this participant, quadriceps tendon Young’s modulus was increased due to osteophytes compressing the quadriceps tendon. In such a case, the prediction formula in this study could not be used. Second, we did not measure muscle activity during gait. It is reported that muscle forces around the hip, knee, and ankle joints affect the maximum knee flexion angle [26]. If these muscle forces were measured, the *β* of Young’s modulus would fluctuate. Third, it was not demonstrated that decreasing quadriceps tendon Young’s modulus by therapeutic intervention increased the maximum knee flexion angle in the swing phase, because this was not examined experimentally. Fourth, this study also indicated that quadriceps tendon Young’s modulus was related to gait speed and step length. However, it was not clear whether the quadriceps tendon Young’s modulus affected gait speed and step length. Fifth, wearing footwear can affect gait parameters related to foot clearance [27] and the relationship shown in this study. Considering these points, further research is necessary.

## 5. Conclusions

Quadriceps tendon Young’s modulus is a predictor for the maximum knee flexion angle in the swing phase of gait in patients with severe knee OA. Clinically, decreasing Young’s modulus may help to increase the maximum knee flexion angle in the swing phase in severe knee OA.

## Figures and Tables

**Table 1:** Participants’ physical characteristics.

Physical Characteristics	Knee OA (n = 30)
Age (years)	75.0 (67.5–76.0) ^2^
Sex (men/women)	10/20
Height (m)	1.54 ± 0.09 ^1^
Weight (kg)	61.0 (48.8–65.9) ^2^
BMI (kg/m^2^)	24.9 (22.1–29.1) ^2^
FTA (°)	181.0 (178.5–184.0) ^2^
KL grade (3/4)	12/18

^1^ Values are presented as a mean ± standard deviation. ^2^ Values are presented as a median (interquartile range). Abbreviations: OA = osteoarthritis, BMI = body mass index, FTA = femorotibial angle, KL = Kellgren–Lawrence.

**Table 2:** Participants’ measured values.

Measurement Values	Knee OA (n = 30)
Active extension angle (°)	−4.3 ± 8.5 ^1^
Active flexion angle (°)	124.1 ± 13.0 ^1^
Quadriceps tendon Young’s modulus (kPa)	271.2 (198.9–424.8) ^2^
Gait speed (m/s)	0.81 ± 0.21 ^1^
Cadence (step/min)	103.7 ± 15.0 ^1^
Step length (m)	0.46 ± 0.08 ^1^
Maximum knee flexion angle in the swing phase (°)	54.9 (48.7–59.5) ^2^
Pain during gait (points)	25.0 (1.5–61.3) ^2^

^1^ Values are presented as a mean ± standard deviation. ^2^ Values are presented as a median (interquartile range). Abbreviations: OA = osteoarthritis.

**Table 3:** Multiple regression analysis results.

Variables	*B*	*95% CI of B*	*p*-Values	*β*	*VIF*
Constant	45.109	25.968–64.249	<0.001 *		
Quadriceps tendon Young’s modulus (kPa)	−0.035	−0.062–−0.008	0.013 *	−0.410	1.391
Gait speed (m/s)	22.367	6.100–38.634	0.009 *	0.433	1.391

* *p* < 0.05. Abbreviations: *B* = partial regression coefficient, *CI* = confidence intervals, *β* = standardized partial regression coefficient, *VIF* = variance inflation factor.

**Table 4:** Correlation coefficients between quadriceps tendon Young’s modulus and maximum knee flexion angle in the swing phase and other measurement values.

	Quadriceps Tendon Young’s Modulus		Maximum Knee Flexion Angle in the Swing Phase	
	*r_s_*	*p*-Value	*r_s_*	*p*-Value
Active extension angle	−0.222	0.238	−0.007	0.971
Active flexion angle	−0.557	0.001 *	0.325	0.079
Quadriceps tendon Young’s modulus	-	-	−0.559	0.001 *
Gait speed	−0.426	0.019 *	0.532	0.002 *
Cadence	−0.252	0.179	0.503	0.005 *
Step length	−0.508	0.004 *	0.457	0.011 *
Maximum knee flexion angle in the swing phase	−0.559	0.001 *	-	-
Pain during gait	0.145	0.445	−0.285	0.126

* *p* < 0.05. Abbreviation: *r_s_* = spearman’s rank correlation coefficient.
